# Olanzapine plus aprepitant, palonosetron, and dexamethasone for nausea and vomiting in patients with breast cancer receiving anthracycline: A retrospective study

**DOI:** 10.1038/s41598-018-34618-x

**Published:** 2018-11-02

**Authors:** Hitoshi Kawazoe, Ryuji Uozumi, Akari Murakami, Michiko Yamashita, Kana Kobayashi-Taguchi, Erina Kusakabe, Haruna Yamasawa, Yoshihiro Yakushijin, Tomonori Nakamura, Yoshiaki Kamei

**Affiliations:** 1 Division of Pharmaceutical Care Sciences, Center for Social Pharmacy and Pharmaceutical Care Sciences, Keio University Faculty of Pharmacy, 1-5-30 Shibakoen, Minato-ku, Tokyo Japan; 2 Division of Pharmaceutical Care Sciences, Keio University Graduate School of Pharmaceutical Sciences, 1-5-30 Shibakoen, Minato-ku, Tokyo Japan; 3 Department of Biomedical Statistics and Bioinformatics, Kyoto University Graduate School of Medicine, 54 Kawahara-cho, Shogoin, Sakyo-ku, Kyoto Japan; 4 Breast Center, Ehime University Hospital, Shitsukawa, Toon, Ehime Japan; 5 Cancer Center, Ehime University Hospital, Shitsukawa, Toon, Ehime Japan

## Abstract

This study aimed to compare the antiemetic efficacy and safety of a four-drug combination with those of a standard three-drug combination in Japanese patients with breast cancer treated with anthracycline. We retrospectively analyzed data from Japanese patients with breast cancer, who had received their first cycle of anthracycline and were treated with aprepitant, palonosetron, and dexamethasone with or without olanzapine. This retrospective observational study was performed at Ehime University Hospital using the electronic medical records. Multivariable and propensity score-adjusted analyses were performed to compare the onset of complete response (CR) failure between the groups. One-hundred and thirty patients were included in this study and the four- and three-drug group had 22 and 108 patients, respectively. Similar to multivariable logistic regression analysis, propensity-adjusted logistic regression analysis revealed that the four-drug group was markedly associated with a decreased odds of CR failure in the overall, acute, and delayed phases (odds ratio [OR]: 0.27, 95% confidence interval [CI]: 0.10–0.73; OR: 0.28, 95% CI: 0.10–0.76; and OR: 0.15, 95% CI: 0.04–0.57, respectively). Additionally, treatment-related adverse events were well tolerated in both the groups. These findings suggest that the antiemetic efficacy of the four-drug combination is superior to that of the standard three-drug combination.

## Introduction

Breast cancer is a major cause of morbidity and mortality in women worldwide. Anthracycline and cyclophosphamide are generally used for patients with breast cancer in neoadjuvant, adjuvant, or palliative settings^1–3^. Anthracycline-based chemotherapy is categorized as a highly emetogenic chemotherapy (HEC), according to multiple national and international antiemetic guidelines issued by the Japanese Society of Clinical Oncology (JSCO), American Society of Clinical Oncology (ASCO), National Comprehensive Cancer Network (NCCN), and Multinational Association of Supportive Care in Cancer (MASCC)/European Society of Medical Oncology (ESMO)^4–7^. Chemotherapy-induced nausea and vomiting (CINV) is associated with a significant deterioration of the patients’ quality of life (QOL)^8^. Antiemetic guidelines recommend a three- or four-drug combination, as the standard antiemetic treatment for CINV in patients receiving HEC, comprising a neurokinin 1 receptor antagonist (NK_1_ RA), a 5-hydroxytryptamine-3 receptor antagonist (5-HT_3_ RA), and dexamethasone, with or without olanzapine^4–7^.

Olanzapine, an atypical antipsychotic drug is called a multi-acting receptor-targeted agent (MARTA) and targets dopaminergic D_1_, D_2_, D_3_, and D_4_ receptors; serotonergic 5-HT_2A_, 5-HT_2B_, 5-HT_2C_, 5-HT_3_, and 5-HT_6_ receptors; adrenergic α_1_ receptor; histamine H_1_ receptor; and several muscarinic receptor subtypes^9–11^. It is hypothesized that blocking of the 5-HT_2C_ receptor directly contributes to the improvement of CINV^12^. Additionally, MARTA-induced weight gain is a frequent adverse event, and enhanced ghrelin release or signaling is considered to underlie MARTA-induced appetite stimulation that indirectly improves CINV^13,14^. Palonosetron, a second-generation 5-HT_3_ RA with a long half-life, is commonly used for HEC or moderately emetogenic chemotherapy as the preferred 5-HT_3_ RA in Japan^4,15,16^. Several pivotal phase III trials have revealed that a three-drug combination is superior to other antiemetic regimens in cancer patients who received HEC^16–18^. However, the antiemetic control of anthracycline remains an unresolved issue in clinical practice^16–19^. A recent phase III trial demonstrated that a four-drug combination of a NK_1_ RA, a 5-HT_3_ RA, dexamethasone, and olanzapine (10 mg) was superior to a standard three-drug combination without olanzapine, for CINV induced by cisplatin and anthracycline^20^. Additionally, a recent phase II dose-finding study demonstrated that olanzapine (5 mg) in a four-drug combination was more suitable for Japanese patients receiving cisplatin^21^. However, the therapeutic benefits of olanzapine (5 mg) in a four-drug combination have not been established in anthracycline-induced CINV.

Therefore, the aim of this study was to compare the antiemetic efficacy and safety of a four-drug combination of olanzapine, aprepitant, palonosetron, and dexamethasone with those of a standard three-drug combination in Japanese patients with breast cancer receiving anthracycline.

## Results

### Patient characteristics

**Figure 1:**
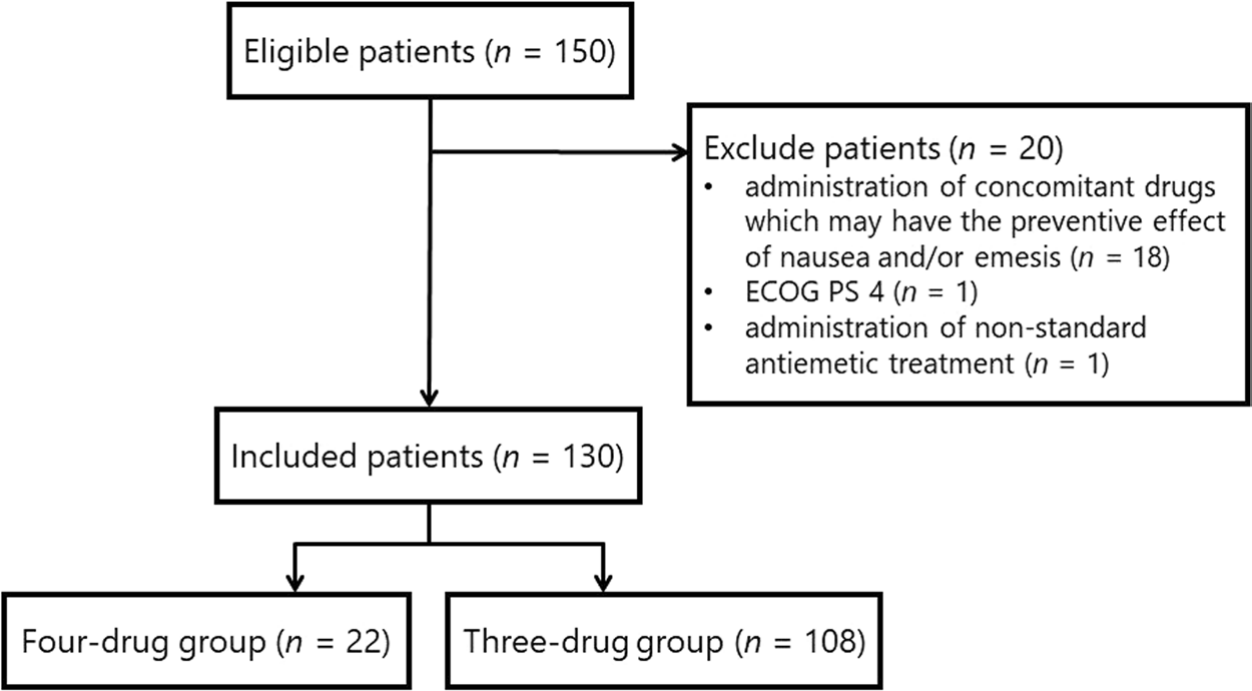
CONSORT flow diagram. ECOG: Eastern Cooperative Oncology Group, PS: performance status.

The CONSORT flow diagram for the 150 patients treated with anthracycline-based chemotherapy regimens is shown in Fig. 1. Based on the eligible and exclusion criteria, 20 patients were withdrawn from the analysis, including 18 patients who had concomitantly used drugs (other than standard antiemetics) that may have prevented nausea and/or emesis during the investigation period. Further, the excluded 18 patients took either a typical antipsychotic, atypical antipsychotic, an antidepressant, and corticosteroid drugs, one patient had Eastern Cooperative Oncology Group performance status (ECOG PS) = 4, and one patient did not receive any standard antiemetic. No other patients met the other exclusion criteria. Thus, 130 patients were assessed for their eligibility in this study. Each patient received the standard full dose of the anthracycline-based chemotherapy. Among these patients, 22 (16.9%) received olanzapine (the four-drug group) and the others (83.1%) did not receive olanzapine (a three-drug group).

Baseline patient characteristics are listed in Table 1. All patients in this study were females and a majority of had ECOG PS = 0. The median age in the four- and three-drug group was 51 years (interquartile range [IQR]: 45–55) and 55 years (IQR: 44–61), respectively. Median body mass index (BMI) for the four- and three-drug group was 22.1 kg/m^2^ (IQR: 20.6–25.3) and 23.3 kg/m^2^ (IQR: 20.5–26.9), respectively. Seven (31.8%) and 39 (36.1%) patients had a history of alcohol consumption in the four- and three-drug group, respectively. In total, the number of patients who received AC, EC, and FEC regimens was 22 (100%), 0 (0%), and 0 (0%), in the four-drug group and 53 (49.1%), 5 (4.6%), and 50 (46.3%) in the three-drug group, respectively.

**Table 1:** Baseline patient characteristics.

		Four-drug group (*n* = 22)	Three-drug group (*n* = 108)
Age (years), median [IQR]		51 [45–55]	55 [44–61]
Sex, *n* (%)	Female	22 (100)	108 (100)
	Male	0 (0)	0 (0)
Height (cm), median [IQR]		157.0 [155.0–163.4]	156.3 [153.0–160.2]
Weight (kg), median [IQR]		56.3 [52.2–63.2]	56.4 [50.5–63.2]
Body surface area (m^2^), median [IQR]		1.59 [1.52–1.69]	1.56 [1.49–1.64]
BMI (kg/m^2^), median [IQR]		22.1 [20.6–25.3]	23.3 [20.5–26.9]
ECOG PS, *n* (%)	0	22 (100)	103 (95.4)
	1	0 (0)	5 (4.6)
Stage, *n* (%)	I	5 (22.7)	10 (9.3)
	II	12 (54.5)	62 (57.4)
	III	5 (22.7)	30 (27.8)
	IV	0 (0)	4 (3.7)
	Recurrence	0 (0)	2 (1.9)
History of smoking habit, *n* (%)	Yes	5 (22.7)	30 (27.8)
	No	17 (77.3)	78 (72.2)
History of alcohol habit, *n* (%)	Yes	7 (31.8)	39 (36.1)
	No	15 (68.2)	69 (63.9)
Anthracycline, *n* (%)	AC	22 (100)	53 (49.1)
	EC	0 (0)	5 (4.6)
	FEC	0 (0)	50 (46.3)

IQR, interquartile range; BMI, body mass index; ECOG, Eastern Cooperative Oncology Group; PS, performance status; AC, doxorubicin and cyclophosphamide; EC, epirubicin and cyclophosphamide; FEC, epirubicin, cyclophosphamide, and 5-fluorouracil.

### Efficacy

**Figure 2:**
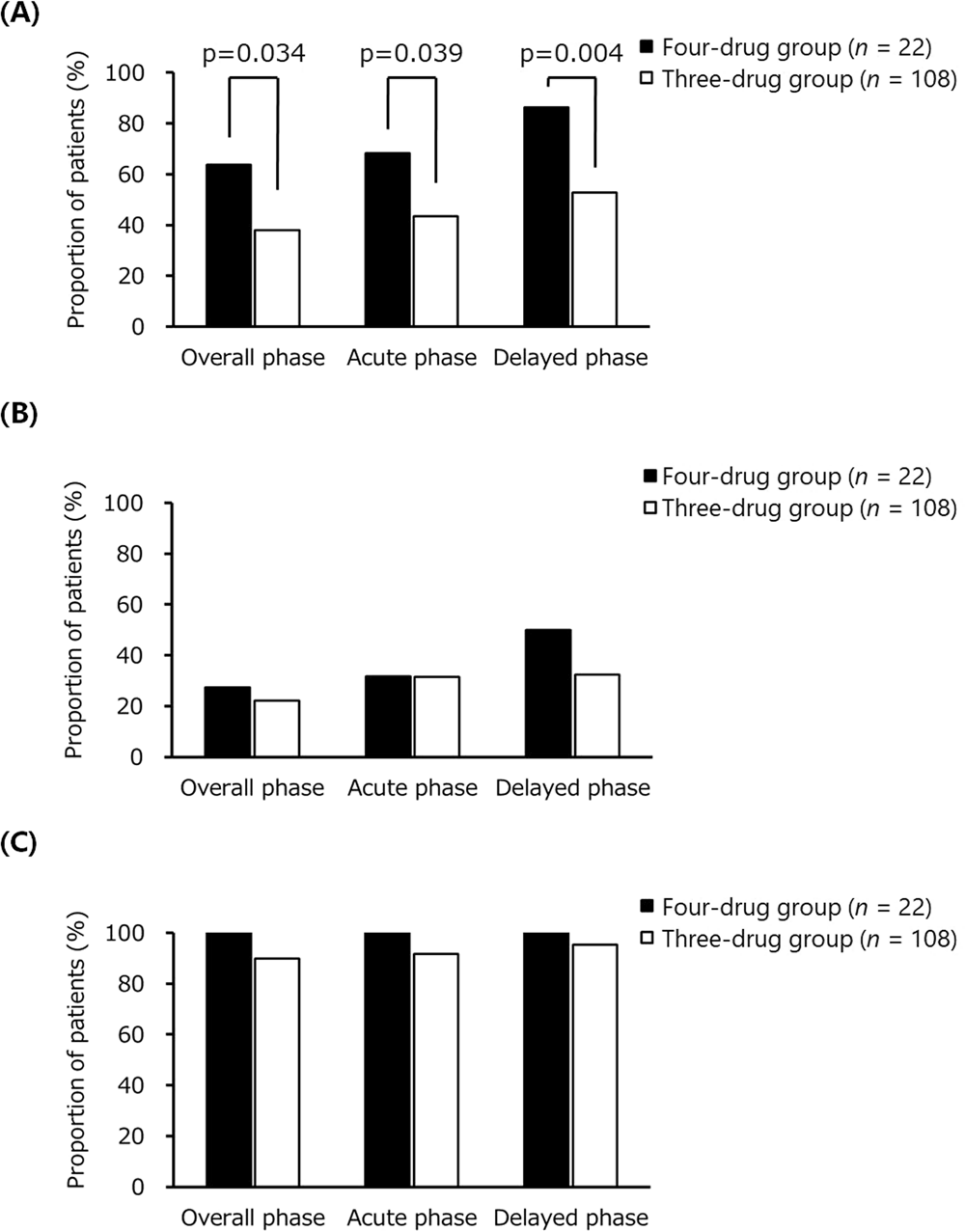
Primary and secondary endpoint of CINV in patients who received the first cycle of anthracycline-based chemotherapy. (**A**) Complete response, (**B**) No nausea, and (**C**) No vomiting. Overall, acute, and delayed phases are the periods of 0–120 hours, within 24 hours of chemotherapy, and 24–120 hours after chemotherapy, respectively. Fisher’s exact test was used to compare the categorical data between both the groups.

The primary and secondary endpoints of CINV are shown in Fig. 2. The proportion of patients in the four-drug group who had complete response (CR) in the overall, acute, and delayed phases was 63.6%, 68.2%, and 86.4%, respectively. This improvement was remarkably higher than that in the three-drug group that exhibited a CR of 38.0%, 43.5%, and 52.8% in the overall, acute, and delayed phase, respectively (Fig. 1A). In a subgroup analysis except for FEC regimen (n = 50), the proportion of patients in the three-drug group who had CR in the overall, acute, and delayed phases was 36.2%, 39.7%, and 53.4%, respectively (data not shown). The primary endpoint was almost similar with or without FEC regimen in the three-drug group. The proportion of patients in the four-drug group who had no nausea in the overall, acute, and delayed phases was 27.3%, 31.8%, and 50.0%, respectively, and was similar to that in the three-drug group (22.2%, 31.5%, and 32.4%, respectively) (Fig. 1B). None of the patients in the four-drug group had any vomiting in the overall, acute, and delayed phase. Similarly, in the three-drug group, the proportion of patients without vomiting in the overall, acute, and delayed phase was 89.8%, 91.7%, and 95.4%, respectively (Fig. 1C). The proportion of patients in the four-drug group who took any domperidone as a rescue in the overall, acute, and delayed phases was 36.4%, 31.8%, and 13.6%, respectively (data not shown), and was remarkably lower than that in the three-drug group (62.0%, 56.5%, and 47.2%, respectively). That is, a CR in the four-drug group was completely associated with no use of rescue medication, because the proportion of patients without vomiting was 100%. Interestingly, the control of nausea was poor, even though vomiting was well controlled in both the groups.

Univariable, multivariable, and inverse probability of treatment weighting (IPTW)-adjusted logistic regression analyses are shown in Table 2. Univariable logistic regression analysis revealed that the four-drug group was greatly associated with a decreased odds of CR failure in the overall, acute, and delayed phases (unadjusted odds ratio [OR]: 0.35, 95% confidence interval [CI]: 0.14–0.91, *p* = 0.030; unadjusted OR: 0.36, 95% CI: 0.14–0.95, *p* = 0.040; and unadjusted OR: 0.18, 95% CI: 0.05–0.63, *p* = 0.008, respectively). Similarly, multivariable logistic regression analysis revealed that the four-drug group was associated with a decreased odds of CR failure in the overall, acute, and delayed phases (adjusted OR: 0.22, 95% CI: 0.07–0.65, *p* = 0.006; adjusted OR: 0.22, 95% CI: 0.07–0.68, *p* = 0.008; and adjusted OR: 0.13, 95% CI: 0.03–0.49, *p* = 0.003, respectively). Furthermore, IPTW-adjusted logistic regression analysis also revealed that the four-drug group was associated with a decreased odds of CR failure in the overall, acute, and delayed phases (adjusted OR: 0.27, 95% CI: 0.10–0.73, *p* = 0.009; adjusted OR: 0.28, 95% CI: 0.10–0.76, *p* = 0.013; and adjusted OR: 0.15, 95% CI: 0.04–0.57, *p* = 0.005, respectively).

**Table 2:** Univariable, multivariable, and IPTW logistic regression analyses of CR failure.

	*n*	No. of events	(%)	Univariable analysis			Multivariable analysis			IPTW-adjusted analysis		
				Unadjusted OR	95% CI	*p*-value	Adjusted OR	95% CI	*p*-value	Adjusted OR	95% CI	*p*-value
(A) Overall phase												
Antiemetics												
Four-drug	22	8	36.4	0.35	(0.14–0.91)	0.030	0.22	(0.07–0.65)	0.006	0.27	(0.10–0.73)	0.009
Three-drug	108	67	62.0	1.00			1.00			1.00		
Age (10 years)	—	—	—	0.66	(0.47–0.93)	0.017	0.60	(0.41–0.88)	0.009			
BMI (1 kg/m^2^)	—	—	—	0.90	(0.83–0.98)	0.019	0.86	(0.78–0.95)	0.002			
History of alcohol habit												
Yes	46	21	45.7	0.47	(0.22–0.97)	0.041	0.30	(0.13–0.70)	0.005			
No	84	54	64.3	1.00			1.00					
(B) Acute phase												
Antiemetics												
Four-drug	22	7	31.8	0.36	(0.14–0.95)	0.040	0.22	(0.07–0.68)	0.008	0.28	(0.10–0.76)	0.013
Three-drug	108	61	56.5	1.00			1.00			1.00		
Age (10 years)	—		—	0.53	(0.37–0.76)	0.001	0.47	(0.32–0.71)	<0.001			
BMI (1 kg/m^2^)	—		—	0.91	(0.83–0.99)	0.025	0.87	(0.79–0.96)	0.006			
History of alcohol habit												
Yes	46	21	45.7	0.66	(0.32–1.36)	0.262	0.44	(0.19–1.03)	0.060			
No	84	47	56.0	1.00			1.00					
(C) Delayed phase												
Antiemetics												
Four-drug	22	3	13.6	0.18	(0.05–0.63)	0.008	0.13	(0.03–0.49)	0.003	0.15	(0.04–0.57)	0.005
Three-drug	108	51	47.2	1.00			1.00			1.00		
Age (10 years)	—		—	0.93	(0.68–1.29)	0.678	0.90	(0.64–1.27)	0.549			
BMI (1 kg/m^2^)	—		—	0.91	(0.83–1.00)	0.040	0.88	(0.80–0.96)	0.007			
History of alcohol habit												
Yes	46	15	32.6	0.56	(0.26–1.18)	0.128	0.38	(0.17–0.89)	0.025			
No	84	39	46.4	1.00			1.00					

IPTW, inverse probability of treatment weighting, OR, odd ratio; CI, confidence interval; BMI, body mass index.

Univariable, multivariable, and IPTW-adjusted logistic regression analyses were used to evaluate an antiemetic efficacy associated with CR failure.

### Safety evaluation

Treatment-related adverse events for both the groups are shown in Table 3. The most common treatment-related adverse event was fatigue (77.3 vs. 86.1%, four-vs. three-drug group, respectively). The incidence of somnolence in the four-drug group was markedly higher than that in the three-drug group (22.7 vs. 1.9%), whereas that of anorexia in the four-drug group was greatly lower than that in the three-drug group (50.0 vs. 77.8%). In addition, the incidence of dexamethasone-induced insomnia in the four-drug group was slightly lower than that in the three-drug group (27.3 vs. 38.0%). The other treatment-related adverse events were common in both the groups. There were two grade 3 treatment-related adverse events (insomnia and ALT increased) in the four-drug group and nine grade 3 treatment-related adverse events (fatigue, constipation, anorexia, insomnia, dizziness, and diarrhea) in the three-drug group. No grade 4 and 5 treatment-related adverse events were reported. All of the adverse events were well tolerated in both the groups and none led to treatment discontinuation.

**Table 3:** Treatment-related adverse events.

	Four-drug group (*n* = 22), *n* (%)			Three-drug group (*n* = 108), *n* (%)		
	Grade 1	Grade 2	Grade 3	Grade 1	Grade 2	Grade 3
Fatigue	14 (63.6)	3 (13.6)	0 (0)	75 (69.4)	17 (15.7)	1 (0.9)
Constipation	13 (59.1)	4 (18.2)	0 (0)	58 (53.7)	11 (10.2)	1 (0.9)
Anorexia	9 (40.9)	2 (9.1)	0 (0)	70 (64.8)	11 (10.2)	3 (2.8)
Insomnia	4 (18.2)	1 (4.5)	1 (4.5)	28 (25.9)	12 (11.1)	1 (0.9)
Somnolence	5 (22.7)	0 (0)	0 (0)	2 (1.9)	0 (0)	0 (0)
Dizziness	5 (22.7)	0 (0)	0 (0)	17 (15.7)	0 (0)	1 (0.9)
Headache	5 (22.7)	0 (0)	0 (0)	31 (28.7)	0 (0)	0 (0)
ALT increased	3 (13.6)	0 (0)	1 (4.5)	18 (16.7)	5 (4.6)	0 (0)
AST increased	3 (13.6)	1 (4.5)	0 (0)	17 (15.7)	3 (2.8)	0 (0)
Diarrhea	2 (9.1)	2 (9.1)	0 (0)	8 (7.4)	2 (1.9)	2 (1.9)
Abdominal pain	1 (4.5)	0 (0)	0 (0)	7 (6.5)	0 (0)	0 (0)
Palpitations	1 (4.5)	0 (0)	0 (0)	4 (3.7)	0 (0)	0 (0)
Hyperglycemia	1 (4.5)	0 (0)	0 (0)	9 (8.3)	1 (0.9)	0 (0)

ASL, aspartate aminotransferase; ALT, alanine aminotransferase. No grade 4 and 5 treatment-related adverse events were reported.

## Discussion

Limited information exists about the therapeutic benefits of a four-drug combination consisting of olanzapine (5 mg), aprepitant, palonosetron, and dexamethasone in Japanese patients with breast cancer treated with anthracycline, in a clinical practice setting. The primary objective of the present pilot study was to test the hypotheses for any future formal study. We hypothesized that the antiemetic efficacy of the four-drug combination is superior to that of the standard three-drug combination. The present study using multivariable logistic regression analysis and the IPTW method with a propensity score to reduce the impact of indication bias in an observational study, demonstrated the antiemetic efficacy of olanzapine (5 mg) in combination with the standard three-drug antiemetic therapy. The four-drug combination achieved a better CR in the overall, acute, and delayed phases. Importantly, treatment-related adverse events were well tolerated in both the groups. Univariable, multivariable, and IPTW-adjusted logistic regression analyses consistently revealed that the four-drug group was markedly associated with a decreased odds of CR failure in the overall, acute, and delayed phases. Thus, the antiemetic efficacy of adding olanzapine (5 mg) in combination with the standard three-drug antiemetic therapy is reliable for the treatment of anthracycline-based CINV.

In the present study, a CR in the four-drug group was completely associated with no use of rescue medication, because the proportion of patients without vomiting was 100%. The grade, visual analogue scale of nausea, or any QOL-related questionnaires were not able to be assessed due to the retrospective nature of the study. The four-drug combination might decrease the intensity of nausea for which the patients required rescue medication, even though the control of nausea was poor.

To our knowledge, the therapeutic benefit of the four-drug combination has not been previously established for patients with breast cancer receiving anthracycline. The therapeutic recommendations in several national and international antiemetic guidelines are solely guided by the chemotherapy-related emetogenicity. Our findings for the control rates of CR and a decreased odds of CR failure in the four-drug group are consistent with those of Navari *et al*. who demonstrated in patients (n = 380) receiving HEC, that a four-drug combination of olanzapine (10 mg) with an NK_1_ RA, a 5-HT_3_ RA, and dexamethasone was significantly associated with a decreased risk of CR failure in the overall, acute, and delayed phases compared with the standard three-drug combination^20^. In the present study, the rate of CR in the overall, acute, and delayed phases was still poorly controlled compared with cisplatin-based CINV, when treated with a standard three-drug combination^16,22^. These findings imply that the control of anthracycline-based CINV remains a critical unmet medical need. In general, females are more vulnerable to CINV^22–25^, and since most patients with breast cancer are female, they already have a baseline risk factor for CINV. Thus, patients with breast cancer receiving anthracycline are a high-risk population, and according to the antiemetic guidelines by ASCO and NCCN^5,6^, more intensive antiemetics like a four-drug combination of olanzapine with an NK1 RA, a 5-HT_3_ RA, and dexamethasone should be considered to prevent CINV.

Interestingly, another phase III trial with olanzapine (10 mg) showed that somnolence remains a serious issue in 73% of patients^26^. Navari *et al*. also reported that olanzapine (10 mg) induced-sedation was a problematic issue on day 2 (severe in 5% of the patients)^20^. In ASCO and NCCN guidelines for antiemetics^5,6^, 10 mg of olanzapine is recommended as an initial dose for the prophylactic treatment of CINV. In addition, antiemetic guidelines by MASCC/ESMO pay attention to the mild and/or moderate sedation in patients treated with 10 mg olanzapine^7^. Further, NCCN guidelines recommend that a lower dose of olanzapine (5 mg) should be considered for elderly or over-sedated patients. Yanai *et al*. reported that 5 mg olanzapine-induced less somnolence than 10 mg (45.5 vs. 53.3%), in a phase II trial conducted in 153 Japanese patients receiving cisplatin HEC^21^. Additionally, no severe toxic effects with olanzapine (5 mg) were observed in the present study. The appropriate timing of olanzapine administration was unknown because the previous phase III trials did not describe about that in detail^20,26^. Since somnolence tends to occur around peak blood olanzapine concentrations, we scheduled its administration at bedtime. Interestingly, somnolence, the most frequent adverse event of olanzapine, might effectively relieve insomnia induced by dexamethasone (27.3 and 38.0% in the four-and three-drug group, respectively).

The present study has several limitations; the first is its retrospective, observational nature, rather than a prospective study. Second, our data were from a single institution with a limited sample size. Third, due to the retrospective nature of the study, methodologically the two groups were non-matching and non-matching sample size. Although there was a difference of patient characteristics who received anthracycline, the primary endpoint was consequently robust with or without FEC regimen. In NCCN guideline^6^, AC combination defined as any chemotherapy regimen that contains an anthracycline and cyclophosphamide. Although 5-fluorouracil including FEC regimen is categorized low emetic risk^6^, that might be little impact of emetogenic potential in anthracycline. In addition, we could not completely assess the patients’ backgrounds, including any history of motion sickness, pregnancy, and morning sickness with pregnancy; thus, a degree of bias may have been introduced into our results. We also performed a propensity score-adjusted analysis to reduce indication bias of an observational study. The fact that a propensity score-adjusted analysis cannot control unmeasured confounders (like those mentioned above) might have affected the results and is a major limitation. Therefore, large-scale and multicenter studies are necessary to confirm the findings of our study. We have planned a phase II and III randomized controlled trial to demonstrate that the four-drug combination that includes olanzapine (5 mg) is associated with a decreased risk of CR failure in the overall, acute, and delayed phases in Japanese patients with breast cancer receiving anthracycline.

In conclusion, this study is the first to demonstrate that the antiemetic efficacy of a four-drug combination is superior to that of the standard three-drug combination in a clinical setting. Our data provide preliminary information about the Japanese population that can likely be translated to other Asian populations, and further highlight the need for additional research in this area.

## Methods

### Study design and patients

This design was a case-control study. A retrospective observational research was carried out at Ehime University Hospital, a tertiary hospital located at the Ehime prefecture, using data from the electronic medical records of Japanese patients (aged ≥ 20 years) with breast cancer, who had received their first cycle of an anthracycline regimen (however, patients on any other chemotherapy could be enrolled if at least 3 months had passed since the final treatment) between May 2011 and March 2018. We switched the standard antiemetic treatment from a three- to a four-drug combination in patients with breast cancer treated with anthracycline regimens after July 2017. Olanzapine was approved by an application based on public knowledge to the Ministry of Health, Labour and Welfare of Japan on June 2017 as an antiemetic drug. Thus, this antiemetic intervention was prospectively performed, but it was just based on practice. This study defined that the case was four-drug group (between May 2011 and June 2017) and the control was three-drug group (between July 2017 and March 2018) according to just by time of treatment. Patient records were de-identified and analyzed anonymously. The eligible criteria consisted of ECOG PS of 0–2, adequate functionality of the bone marrow, liver, and kidney within 7 days of chemotherapy (absolute neutrophil count ≥ 1,500 cells/mm^3^, aspartate aminotransferase and alanine aminotransferase ≤ 3.0 times the normal upper limit, blood bilirubin ≤ 1.5 times the normal upper limit, and creatinine ≤ 1.5 times the normal upper limit). We retrospectively extracted the necessary clinical and demographic information at baseline including age at the time of treatment, sex, body surface area, BMI, ECOG PS, cancer stage, history of tobacco use and habitual alcohol consumption, comorbidity of diabetes mellitus, chemotherapy regimen and dose, antiemetic use, CINV, use of rescue medication, and other eligibility criteria. This study defined comorbidity of diabetes mellitus as a past or current medical history of diabetes. Additionally, pharmacists at the hospital and community pharmacies routinely confirmed compliance with oral medicines.

Patients who met any of the following criteria were excluded from this study: 1) complications that induced nausea and/or emesis before the initiation of chemotherapy (e.g., obstruction of gastrointestinal tract, symptomatic ulcerative diseases, or brain metastases); 2) administration of concomitant drugs that may prevent nausea and/or emesis during the investigation period, except for standard antiemetic prophylaxis (e.g., typical antipsychotics, atypical antipsychotics, antidepressants, corticosteroids, or dopamine receptor antagonists for any other reason); 3) concomitant radiotherapy at an esophageal site during the investigation period; 4) administration of total parenteral nutrition before initiation of chemotherapy; 5) administration of non-standard antiemetic treatment; 6) uncontrolled diabetes mellitus during the investigation period; and 7) pregnant women.

### Anthracycline-based chemotherapy

All patients received anthracycline-based chemotherapy that consisted of AC regimen: doxorubicin (60 mg/m^2^) and cyclophosphamide (600 mg/m^2^), EC regimen: epirubicin (90 mg/m^2^) and cyclophosphamide (600 mg/m^2^), or FEC regimen: epirubicin (100 mg/m^2^), cyclophosphamide (500 mg/m^2^), and 5-fluorouracil (500 mg/m^2^). The first course of chemotherapy was administered in an inpatient setting, whereas sequential courses were administered in an outpatient setting according to the principles of our institute.

### Antiemetic treatment

On day 1, all patients in the three-drug group received oral aprepitant (125 mg) 60 minutes prior to chemotherapy, followed by intravenous palonosetron (0.75 mg) and dexamethasone (9.9 mg; 12 mg as dexamethasone sodium phosphate) 15 minutes prior to chemotherapy. Next, patients received oral aprepitant (80 mg) and oral dexamethasone (8 mg) on days 2 and 3, and only oral dexamethasone (8 mg) on day 4, in accordance with the national antiemetic guidelines of JSCO^4^. In contrast, all patients in the four-drug group received oral olanzapine (5 mg) at bedtime on days 1–4, in addition to the above mentioned three-drug combination of aprepitant, palonosetron, and dexamethasone. This regimen was based on a recent phase II trial that demonstrated that olanzapine (5 mg) was more suitable for Japanese patients receiving cisplatin^21^. All patients in this study were prescribed oral domperidone (10 mg) as an on-demand rescue medication.

### Endpoint assessment

Clinical assessment of CINV in each patient was routinely performed by healthcare professionals including clinicians, pharmacists, and nurses in the inpatient and outpatient settings. The primary endpoint was the CR (defined as no vomiting and no use of rescue medication) in the overall phase (0–120 hours), acute phase (within 24 hours), and delayed phase (24–120 hours) after chemotherapy. As the secondary endpoint, we evaluated no nausea (no episode) and no vomiting (no episode) in the overall, acute, and delayed phases after chemotherapy. In addition, treatment-related adverse events including physical examination and laboratory findings were also evaluated by clinicians, pharmacists, and nurses from day 1 of chemotherapy to day 21, according to the Common Terminology Criteria for Adverse Events, version 4.0.

### Statistical analysis

Fisher’s exact test was used to compare the categorical data between the groups. We calculated the OR and 95% CI as an indicator of CR failure using univariable and multivariable logistic regression models between the groups. To account for indication bias due to lack of randomization, we performed a propensity score-adjusted analysis, i.e., the IPTW-adjusted analysis^27^. The propensity score of receiving a four-drug group (propensity score) was estimated for each patient using a logistic regression model^28^. The model included the following independent variables: patient’s age, BMI, and a history of habitual alcohol intake. Several previous studies have reported these variables as possible patient-related risk factors of CINV^22–25^. Next, a logistic regression model, adjusting for propensity score with IPTW, was used to compare the onset of CR failure, the primary endpoint, between the four- and three-drug groups. For the purpose of this study, we did not impute any missing data. All statistical analyses were performed using SAS version 9.4 (SAS Institute, Cary, NC, USA).

### Ethics statement

The study protocol was approved by the ethics committee of Ehime University Hospital (approval number: 1804012) and was conducted in accordance with the Declaration of Helsinki and the Ethical Guidelines for Medical and Health Research involving Human Subjects by the Ministry of Education, Culture, Sports, Science and Technology, and the Ministry of Health, Labour and Welfare of Japan. Japanese law does not require individual informed consent from participants in a non-invasive observational trial such as the present study. Therefore, we used our official breast center and clinical research support center website as an opt-out method rather than acquiring written or verbal informed consent from the patients.

## Data Availability

All data generated or analyzed during this study are included in this published article.
